# Heparan sulfates facilitate harmless amyloidogenic fibril formation interacting with elastin-like peptides

**DOI:** 10.1038/s41598-018-21472-0

**Published:** 2018-02-15

**Authors:** Federica Boraldi, Pasquale Moscarelli, Brigida Bochicchio, Antonietta Pepe, Anna M. Salvi, Daniela Quaglino

**Affiliations:** 1 Department of Life Sciences, University of Modena and Reggio Emilia, Modena, Italy; 2 Department of Sciences, University of Basilicata, Potenza, Italy

## Abstract

Heparan sulfates (HSs) modulate tissue elasticity in physiopathological conditions by interacting with various matrix constituents as tropoelastin and elastin-derived peptides. HSs bind also to protein moieties accelerating amyloid formation and influencing cytotoxic properties of insoluble fibrils. Interestingly, amyloidogenic polypeptides, despite their supposed pathogenic role, have been recently explored as promising bio-nanomaterials due to their unique and interesting properties. Therefore, we investigated the interactions of HSs, obtained from different sources and exhibiting various degree of sulfation, with synthetic amyloidogenic elastin-like peptides (ELPs), also looking at the effects of these interactions on cell viability and cell behavior using *in vitro* cultured fibroblasts, as a prototype of mesenchymal cells known to modulate the soft connective tissue environment. Results demonstrate, for the first time, that HSs, with differences depending on their sulfation pattern and chain length, interact with ELPs accelerating aggregation kinetics and amyloid-like fibril formation as well as self-association. Furthermore, these fibrils do not negatively affect fibroblasts’ cell growth and parameters of redox balance, and influence cellular adhesion properties. Data provide information for a better understanding of the interactions altering the elastic component in aging and in pathologic conditions and may pave the way for the development of composite matrix-based biomaterials.

## Introduction

Heparan sulfates (HSs) are anionic polysaccharides made up of repeating amino sugar-uronic acid disaccharide units that undergo a various degree of sulfation^1^. They are widely distributed as membrane and extracellular glycosaminoglycans, playing a number of important biological and pharmacological activities in normal and in pathological conditions mainly due to their ability to interact with a multitude of proteins including matrix components as well as cytokines and growth factors^2^.

Within the extracellular matrix, HSs are well known to interact *in vivo* with tropoelastin (soluble precursor form of elastin), contributing to elastic fiber formation and stability^3–6^, thus modulating tissue elasticity. These findings have been further supported by *in vitro* observation showing that HSs interact with alpha elastin as well as with elastin derived peptides (EDPs), inducing changes in their coacervation temperature and aggregation properties^6–9^. Elastin, being the major constituent of elastic fibers, confers recoil to human tissues undergoing physical stress and repetitive distension (lung, skin, blood vessels, bladder, heart). However, loss of tissue elasticity is the hallmark of increasing age or of a number of pathological conditions, being the consequence of the almost absent elastin turnover, of damages to elastic fibers, as degradation and fragmentation, which release biologically active EDPs^10–13^, or favour the deposition of protein aggregates called elastotic material^14,15^. Although, these aggregates have not been fully characterized, it appears that they consist predominantly of fibronectin, microfibrillar proteins, elastin and amyloid-like proteins^16–18^. Consistently, amyloid deposits were demonstrated in the aged arterial wall, further suggesting that elastin represents a precursor of these aggregates^19^.

Interestingly, HSs have been shown to be present in amyloid deposits *in vivo*^20^, to bind to amyloidogenic peptides *in vitro* and to accelerate the process of amyloidogenesis favouring aggregation, nucleation and formation of intermediates/fibrils rich in β-sheet structures in a characteristic cross-β conformation^21–24^.

Within this context, several studies have shown that, among the EDPs encoded by specific elastin gene sequences, a number of these peptides is characterized by unordered and extended conformations behaving similarly to the whole molecule^25,26^, whereas others exhibit an ordered β-sheet structure, being more insoluble and revealing amyloidogenic properties^27–30^, in agreement with the finding that elastin and elastin fragments are present in amyloid deposits^16–18^. Similar amyloidogenic properties were demonstrated for elastin-like peptides (ELPs) containing the XGGZG sequence, with guest residues constituted of leucine and/or valine repeated motifs^31–33^. The demonstration that these peptides do not exhibit any evidence of toxicity in cell culture^34^ supported the concept that they could be safely used as bio-inspired nanomaterials, similarly to other amyloidogenic peptides characterized by easy production, low cost, outstanding mechanical stability and remarkably regular architecture^35^.

Despite the significant progress made in the last years in understanding amyloidogenic protein interactions with a variety of extracellular matrix-associated biomolecules^36,37^, however no data are available on the ability of HSs to bind to ELPs and if these interactions may influence cell behaviour and/or may induce a cytotoxic effect.

The aim of this study was to investigate the interactions between two HSs, from different sources and with diverse sulfation degree, and ELPs constituted of the valine-glycine-glycine-valine-glycine (VGGVG) or (VGGVG)_3_ sequences in addition to (VGGVG)_n_ and leucine-glycine-glycine-valine-glycine (LGGVG)_n_ obtained by polycondensation reaction. The ability of HSs to influence amyloid-like fibrils’ formation as well as their aggregation kinetics and morphology were examined. Moreover, biological parameters (i.e. cell proliferation, cell death, cell adhesion and redox balance) were investigated in murine Balb/c 3T3 fibroblasts cultured in the presence of ELPs with or without HSs.

## Results

### HSs influence the kinetics of fibril formation

**Figure 1:**
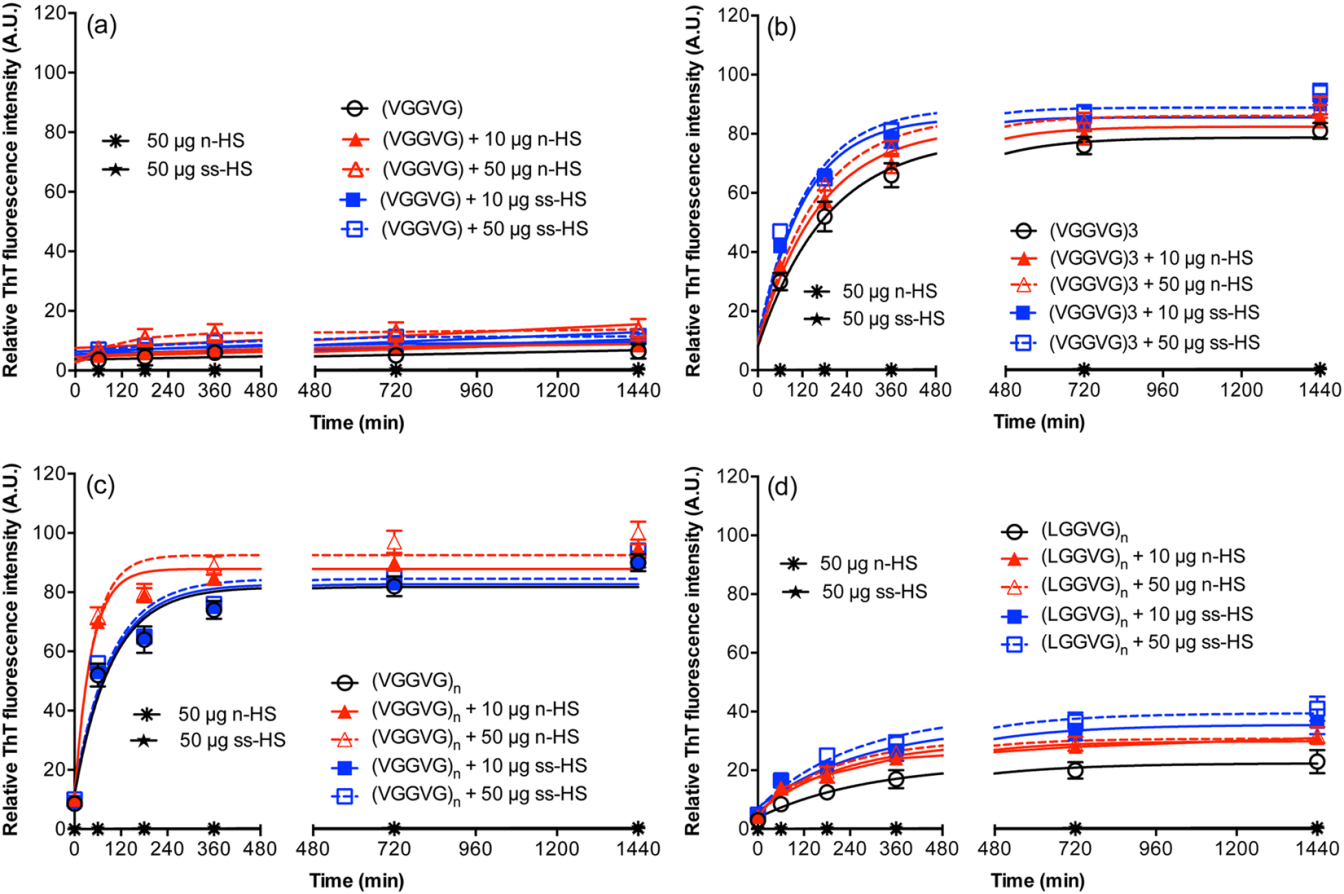
Thioflavin T (ThT) fluorescence for real-time monitoring of fibrillation kinetics. (**a**) (VGGVG), (**b**) (VGGVG)_3_, (**c**) (VGGVG)_n_ and (**d**) (LGGVG)_n_ were incubated in aqueous solution for 24 h at 37 °C in the absence or in the presence of natural (n-HS) or of semisynthetic (ss-HS) heparan sulfates. In each panel, data of ThT assay for n-HS and ss-HS alone (50 μg/mL) are also shown. Data are expressed as the mean ± SD of three experiments.

Kinetics and yield of fibril formation of ELPs solutions, in the absence or presence of two HSs concentrations, were investigated at different time points by measuring the intensity of Thioflavin T (ThT) fluorescence^38^. In Fig. 1 ThT fluorescence curves revealed strong differences in fibrillation kinetics depending on the type of ELPs and HSs, but not on the concentration of the HS used. In particular, ThT fluorescence was closed to zero for HSs alone (Fig. 1), confirming the absence of amyloidogenic fibril formation. Results were independent of HS concentration (data not shown). Negligible values were observed for the monomer (VGGVG), both in the absence or presence of HSs (Fig. 1a and Table 1), indicating insignificant fibrillation. By contrast, when HSs were added to the other peptides (Fig. 1b,c and d and Table 1), the time course of ThT fluorescence was characterized by two phases (Fig. 1): an exponential growth phase corresponding to kinetics of aggregation and a final levelling off, when the process reaches an equilibrium. In particular, apparent rate constants for fibril formation upon addition of natural-heparan sulfate (n-HS) revealed kinetics faster than those of peptides alone (Table 1). With the exception of (VGGVG)_3_, addition of semisynthetic-heparan sulfate (ss-HS) did not show any significant effect on the rate of fibril formation. To be noted, however, that, in the presence of HSs, the fluorescence intensity at aggregation plateau was always higher than that measured with ELPs alone (Fig. 1), suggesting the presence of morphologically different fibrils and/or of a different amount of amyloidogenic intermediates. Interestingly, replacement of valine with leucine in the peptide sequence led to a different aggregation kinetics, as clearly shown for (LGGVG)_n_ *vs* (VGGVG)_n_ (Fig. 1d and c).

**Table 1:** Aggregation rate constants (k_agg_) for elastin-like peptides (ELPs) in presence or in absence of natural (n-HS) or semisynthetic (ss-HS) heparan sulfates.

Condition	k_agg _× 10^−3^ (min^−1^)	t_50_^1^
(VGGVG)_3_	5,327 ± 0,37	130
(VGGVG)_3_ + 10 μg/mL n-HS	6,003 ± 0,47*	115
(VGGVG)_3_ + 50 μg/mL n-HS	6,367 ± 0,42**	109
(VGGVG)_3_ + 10 μg/mL ss-HS	8,066 ± 0,58***	86
(VGGVG)_3_ + 50 μg/mL ss-HS	7,743 ± 0,75***	90
(VGGVG)_n_	10,65 ± 1,57	66
(VGGVG)_n_ + 10 μg/mL n-HS	23,22 ± 2,57****	29
(VGGVG)_n_ + 50 μg/mL n-HS	21,08 ± 2,76****	33
(VGGVG)_n_ + 10 μg/mL ss-HS	10,65 ± 1,50	65
(VGGVG)_n_ + 50 μg/mL ss-HS	10,52 ± 1,72	66
(LGGVG)_n_	3,470 ± 0,74	200
(LGGVG)_n_ + 10 μg/mL n-HS	4,352 ± 0,63*	158
(LGGVG)_n_ + 50 μg/mL n-HS	4,922 ± 0,34***	141
(LGGVG)_n_ + 10 μg/mL ss-HS	3,660 ± 0,54	189
(LGGVG)_n_ + 50 μg/mL ss-HS	3,907 ± 0,60	177
**Condition**	**Equation**	
(VGGVG)	Y = 0,002197X + 3,686	
(VGGVG) + 10 μg/mL n-HS	Y = 0,003511X + 4,608	
(VGGVG) + 50 μg/mL n-HS	Y = 0,005535X + 7,572	
(VGGVG) + 10 μg/mL ss-HS	Y = 0,003450X + 5,475	
(VGGVG) + 50 μg/mL ss-HS	Y = 0,004505X + 6,379	

The rate constants ± standard deviations were calculated through exponential curve fitting. For (VGGVG) ± heparan sulfates linear regression function is reported indicating insignificant fibrillation. t_50_ (=half-time is the amount of time expressed in minutes required to reach half of the maximum fluorescence intensity of the final fluorescence). *p < 0.05; **p < 0.01; ***p < 0.001; ****p < 0.0001 ELP alone *vs* the same EPL + HS.

The contribution of electrostatic interactions between hydrophobic ELPs and HSs was assessed measuring ThT fluorescence in Tris buffer solution. Fluorescence intensity displayed values similar to those obtained in water, either in the absence or in the presence of HSs, indicating that salts did not have any effect on fibril formation by ELPs (see supplementary Fig. S1).

A similar profile was observed measuring the absorbance increase in solution’s turbidity at the end of the ELPs aggregation process in the absence/presence of HSs (data not shown).

### HSs modify the supramolecular organization of elastin-like peptides

Since data from ThT fluorescence assay appeared independent of HS concentration, analyses were performed using ELP ± 10 μg/mL of HSs.

To probe for differences in fibrils’ morphology, transmission electron microscopy (TEM) images of HSs and ELP alone or in combination were acquired after 24 h of incubation (time point corresponding to ThT fluorescence plateaus). In supplementary Fig. S2, negatively stained HSs revealed a globular structure without any significant difference between n-HS and ss-HS.

**Figure 2:**
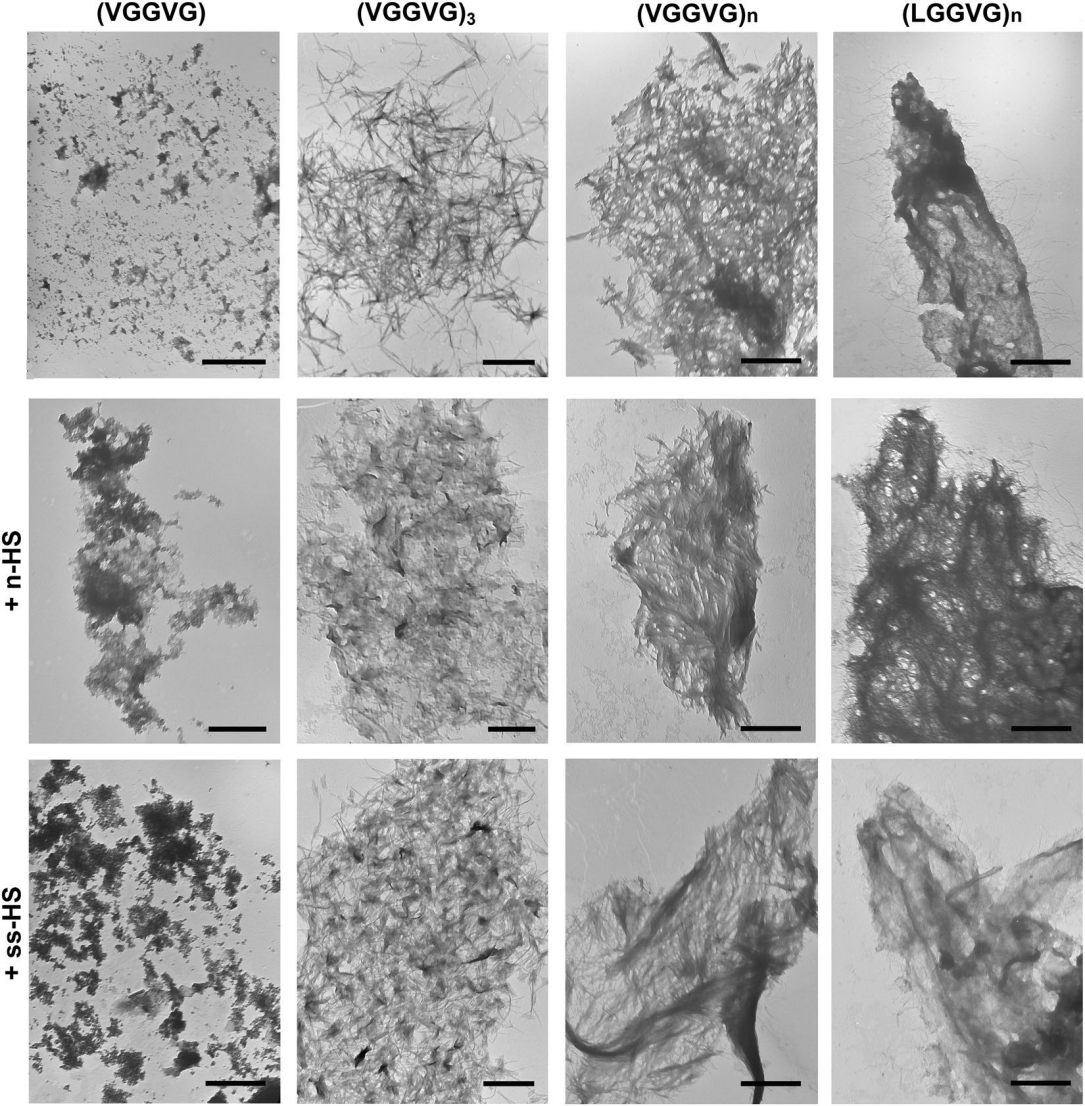
Transmission electron microscopy. Images show (VGGVG), (VGGVG)_3_, (VGGVG)_n_ and (LGGVG)_n_ incubated in water for 24 h at 37 °C in the absence or in the presence of natural (n-HS) or of semisynthetic (ss-HS) heparan sulfates. Samples are negatively stained on formvar coated copper grids. Bars: 1 μm.

The addition of natural and semisynthetic HS to ELPs favoured aggregation and side by side self-association of fibrillar nanostructures compared to ELPs alone. The morphology of fibrillar aggregates was dependent not only on the amino acid sequence, but also on the ability to form supramolecular structures (i.e. peptide length). Consistently, the monomer (VGGVG) never formed fibrillar structures, independently of the presence of HSs (Fig. 2). By contrast, (VGGVG)_3_ self-assembled into short and spatially tangled amyloid-like fibrils, whereas (VGGVG)_n_ gave rise to intertwined, branched and highly visible elongated amyloid fibrils. Interestingly, changes in the amino acid sequence, as in (LGGVG)_n_, induced the formation of several dense aggregates mixed to short and less ordered fibrillar structures (Fig. 2). The presence of HSs seemed to induce a more packed/ordered arrangement of fibrils of (VGGVG)_n_ and (LGGVG)_n_ compared to (VGGVG)_3_ (Fig. 2).

### HSs enhance amyloid-like fibril formation

To visualize the presence of amyloid deposits, bacteriology Petri dishes were coated with HSs alone or with different ELPs, alone or in combination with HSs. After Congo red (CR) staining, plates were observed by light microscopy (see supplementary Fig. S3). Staining of amyloid-like fibrils was clearly visible on surfaces coated with (VGGVG)_3_, (VGGVG)_n_ and (LGGVG)_n_. As expected, staining was absent on plates coated either with n-HS and ss-HS (data not shown) or with the monomer (VGGVG) (see supplementary Fig. S3). HSs addition to ELPs determined a strong staining increase in all conditions with the exception of (VGGVG), since the monomer never forms amyloid-like fibrils^34^.

To quantify amyloid-like fibril formation, CR binding assay was carried out in all experimental conditions after 24 h-incubation. Unlike ThT fluorescence assay, CR staining is not suitable for real-time experiments, since it partially inhibits fibril formation^39^ and therefore the dye has to be added when fibrils are already formed.

**Figure 3:**
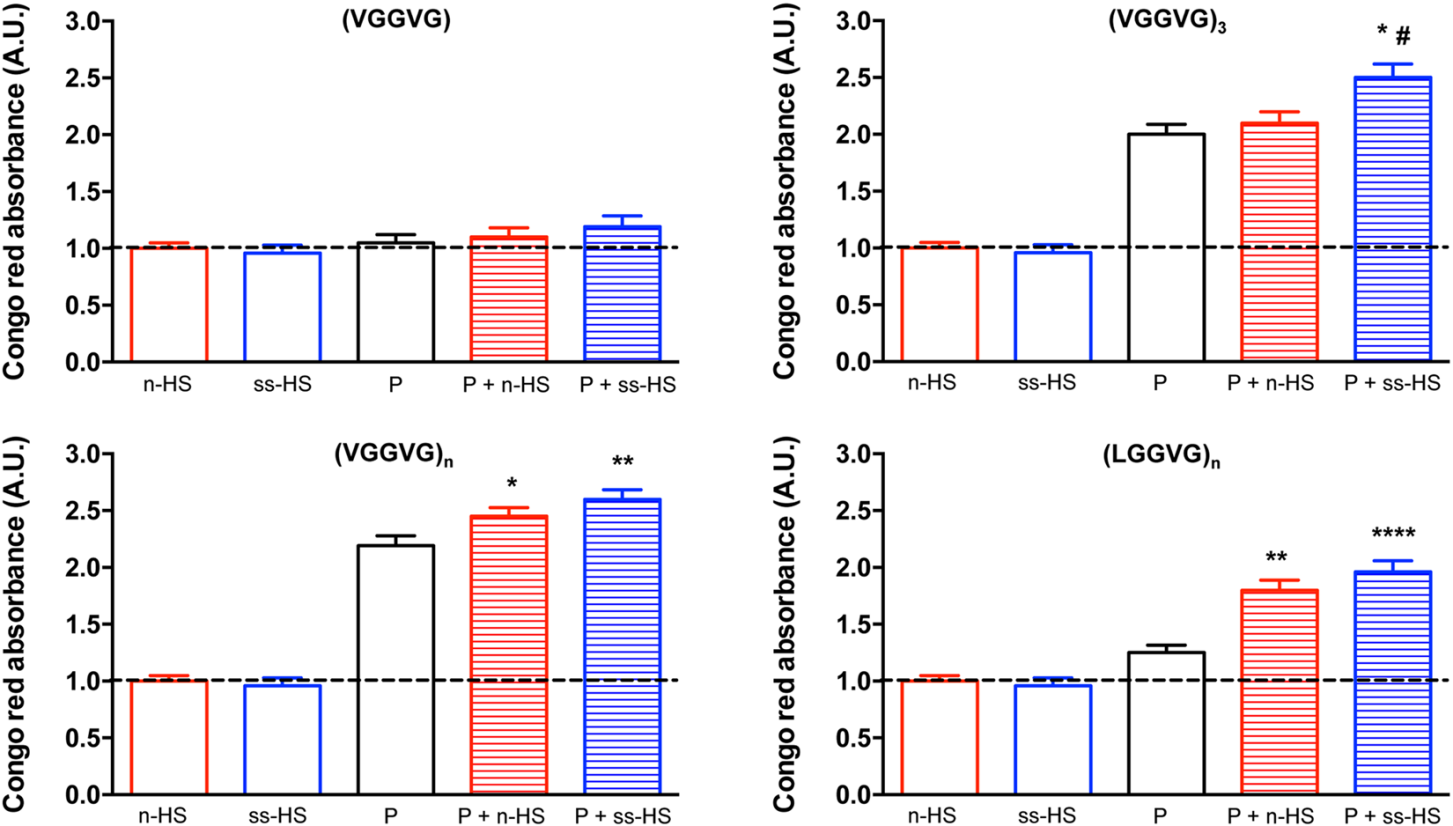
Spectroscopic assay by Congo red (CR). Natural (n-HS) or semisynthetic (ss-HS) heparan sulfate alone or elastin-like peptide (P) alone or their mixture were mantained for 24 h at 37 °C in acqueous solution. Data are normalized to CR background set at 1 (dotted line) and are expressed as the mean ± SD of three experiments. *p < 0.05; **p < 0.01; ****p < 0.0001 peptide *vs* other conditions; ^#^p < 0.05 peptide + n-HS *vs* same peptide + ss-HS.

The monomer and HSs, alone or in combination, showed absorption values at 540 nm similar to basal levels (Fig. 3). Values were higher in all other experimental conditions with an additional positive effect upon addition of HSs (Fig. 3). To be noted that substitution of the first valine of the (VGGVG) sequence with a leucine significantly reduced the amount of β-sheet structures of (LGGVG)_n_ compared to (VGGVG)_n_. Interactions of (LGGVG)_n_ with both HSs significantly increased CR staining, although values remained lower than those observed for the more amyloidogenic (VGGVG)_3_ and (VGGVG)_n_.

### Cell growth and cell viability are not affected by HSs and ELPs

HSs plus ELPs did not inhibit cell growth of Balb/c 3T3 fibroblasts compared to untreated cells (see supplementary Fig. S4). Furthermore, to evaluate the presence of dead cells, cultures were periodically evaluated by light microscopy looking for floating (i.e. detached) cells and by flow cytometry after propidium iodide (PI) staining. This polar fluorescent dye can only enter into cells lacking plasma membrane integrity, thus discriminating between damaged and healthy cells. In all experimental conditions only a small fraction of cells was PI positive (≤4%) (see supplementary Fig. S4). Similarly, the number of cells cultured in the presence of HSs or of peptides alone was similar to that obtained in untreated cells over time in culture (data not shown).

### HSs and ELPs in the cell culture medium do not affect ROS amount

**Figure 4:**
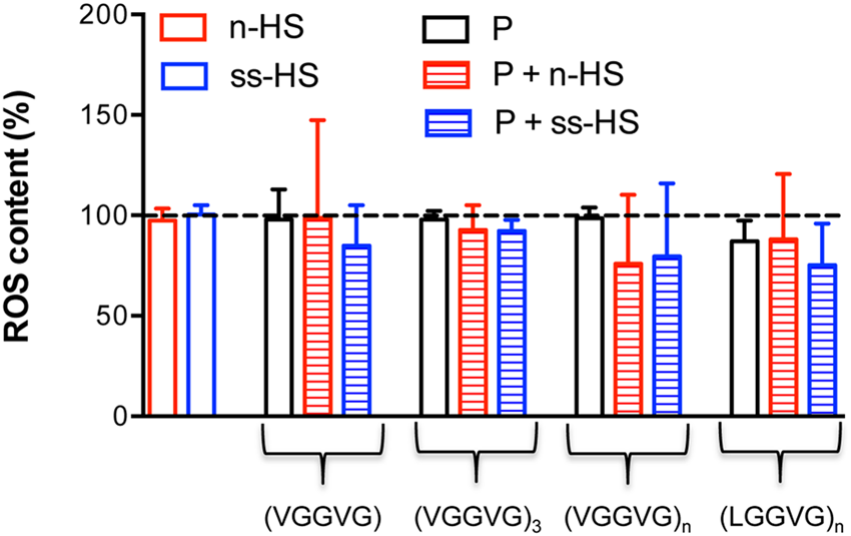
Intracellular content of reactive oxygen species (ROS) evaluated by flow cytometry. The fluorescent probes H_2_DCF-DA and DH_2_ were used to detect hydrogen peroxide (H_2_O_2_) and superoxide anion (O_2_^•−^), respectively. Cells were incubated with natural (n-HS) or semisynthetic (ss-HS) heparan sulfate alone, or with peptides (P) i.e. (VGGVG), (VGGVG)_3_, (VGGVG)_n_ and (LGGVG)_n_ alone or in combination with HSs. Values are expressed as percentage of H_2_O_2_ + O_2_^•−^ normalized to ROS content obtained in cells cultured in standard medium (dotted line set at 100). Data are represented as mean values ± SD of three experiments.

The intracellular presence of the two most representative reactive oxygen species (ROS), i.e. hydrogen peroxide and anion superoxide, was measured by flow cytometry in cultured Balb/c 3T3 fibroblasts upon loading with specific fluorescent probes. The amount of ROS (O_2_^•−^+H_2_O_2_) that can be detected in untreated cells was not modified by HSs alone, neither by ELPs alone or supplemented with natural or semisynthetic heparan sulfates (Fig. 4).

### Adhesion of Balb/c 3T3 fibroblasts is modified on surfaces coated with HSs and ELPs

An adhesion assay was performed using Balb/c 3T3 fibroblasts seeded on uncoated or on coated plates. Table 2 shows the percentage of substrate-adherent cells in all experimental conditions compared to the positive control (i.e. cells seeded on tissue culture plates). Results similar to the positive control were noticed only with n-HS and ss-HS alone or with the fibrillar (VGGVG)_3_ (Table 2). Cells seeded on plates coated with the other ELP exhibited increased adhesion compared to uncoated surfaces, but cell adhesion in these experimental conditions was always reduced compared to the positive control or to HS-coated surfaces.

**Table 2:** Adhesion assay on bacteriology Petri dishes uncoated or coated.

	30 min	60 min	180 min	360 min	p values^a^				
Negative control (BSA coated)	6,20 ± 0,12	6,28 ± 1,13	6,25 ± 0,56	6,91 ± 0,67	°	♯	^•^	◊	
Uncoated	31,39 ± 3,30	38,57 ± 6,87	43,04 ± 5,97	45,44 ± 1,19		♯	^•^	◊	
Positive control	67,57 ± 2,22	74,40 ± 2,39	95,36 ± 3,28	**100 **±** 5,09**	°				
n-HS	65,12 ± 9,33	65,19 ± 8,88	98,25 ± 7,00	103,0 ± 12,0	°				
ss-HS	62,54 ± 9,85	70,97 ± 14,0	94,81 ± 5,61	97,00 ± 3,94	°				
(VGGVG)	54,93 ± 2,21	62,78 ± 5,07	71,30 ± 3,17	73,80 ± 3,38	°	♯	^•^	◊	
(VGGVG) + n-HS	36,71 ± 2,31	46,34 ± 1,25	49,77 ± 2,30	52,46 ± 3,28	°	♯	^•^	◊	*
(VGGVG) + ss-HS	36,02 ± 2,39	43,19 ± 2,69	47,38 ± 2,69	49,77 ± 3,50	°	♯	^•^	◊	*
(VGGVG)_3_	68,75 ± 4,12	86,39 ± 5,10	92,26 ± 4,65	97,34 ± 3,14	°				
(VGGVG)_3_ + n-HS	59,79 ± 3,02	74,14 ± 8,23	82,51 ± 4,36	84,90 ± 1,25	°	♯	^•^	◊	*
(VGGVG)_3_ + ss-HS	55,60 ± 1,25	71,59 ± 2,99	78,77 ± 5,20	82,51 ± 0,99	°	♯	^•^	◊	*
(VGGVG)_n_	58,75 ± 3,45	68,47 ± 4,08	74,47 ± 3,81	76,42 ± 3,12	°	♯	^•^	◊	
(VGGVG)_n_ + n-HS	50,82 ± 4,25	60,24 ± 7,01	66,81 ± 2,99	69,51 ± 6,15	°	♯	^•^	◊	
(VGGVG)_n_ + ss-HS	46,70 ± 1,27	56,80 ± 2,93	61,43 ± 3,58	66,76 ± 3,28	°	♯	^•^	◊	*
(LGGVG)_n_	57,22 ± 3,76	64,31 ± 1,69	69,53 ± 3,26	72,65 ± 3,66	°	♯	^•^	◊	
(LGGVG)_n_ + n-HS	48,87 ± 2,12	53,21 ± 1,49	57,55 ± 3,88	63,38 ± 2,69	°	♯	^•^	◊	*
(LGGVG)_n_ + ss-HS	43,95 ± 2,69	50,97 ± 4,23	53,51 ± 2,39	59,19 ± 3,28	°	♯	^•^	◊	*

The percentage of adherent cells after 30, 60, 180 and 360 minutes compared to positive control (tissue culture plates) at 360 min set at 100. Data are expressed as mean ± SD of three experiments performed at least in triplicate. Differences were considered significant for p < 0.05.

a) °All conditions *vs* uncoated; ^#^All conditions *vs* positive control; ^•^All conditions *vs* n-HS alone; ^◊^All conditions *vs* ss-HS alone.

*ELP + n-HS/ss-HS *vs* the same ELP alone. ^a^p values reflect time dependent variations of cellular adhesion analysed by hyperbolic curve. Best fit values, standard errors and degrees of freedom obtained from each condition were compared using one way analysis of variance (ANOVA) followed by post hoc Tukey testing.

Cell adhesion on surfaces coated with all ELPs + ss-HS was significantly reduced in comparison with ELP alone and/or HS alone (Table 2 and Supplementary Fig. S5). In the case of ELP + n-HS (Table 2 and Supplementary Fig. S5), the effects seemed dependent on the type of ELP. In particular, (VGGVG)_n_ + n-HS did not significantly modify the number of adherent cells compared to fibrillar ELP alone. Adhesion on coated surfaces with (VGGVG), (VGGVG)_3_, and (LGGVG)_n_ + n-HS was further reduced in comparison with ELP alone. This reduction is not indicative of a poor affinity of cells to these amyloid fibrils, since fibroblasts are clearly spread onto CR-positive amyloid fibrillar material (Supplementary Fig. S6). As expected, cell attachment was inhibited in the negative control, i.e. serum bovine albumin (BSA) coated plates.

## Discussion

Amyloidogenesis has been implicated in a broad spectrum of diseases caused by accumulation of abnormally folded and aggregated proteins in many different tissues^36^. Nevertheless, a question has been recently raised wether toxicity of amyloid fibrils is related to the composition of the fibrils or to the beta-sheet structural organization of the fibrils *per se*^35^.

Interestingly, amyloid deposits are frequently found in ageing connective tissues, suggesting that elastin represents a precursor of these aggregates^19^. Within the extracellular matrix, heparan sulfates (HSs) are well known to interact with elastin^3–6^ and with elastin-derived peptides^6–9^, possibly modulating tissue elasticity, but HSs have been also found to be associated with amyloid deposits *in vivo*^20,40^, and have been shown to be involved in amyloid fibril formation of many peptides and proteins *in vitro*^21,24,41^. HSs, along with a variety of other molecules, exhibit amyloid promoting activity by inducing the conformational transition required for fibril formation and by enhancing lateral aggregation of small fibrils^41,42^.

The present study has been undertaken with the aim to demonstrate if HSs, obtained from different sources and exhibiting various degree of sulfation, can interact with synthetic amyloidogenic elastin-like peptides (ELPs) based on the (XGGVG) sequence, where G = glycine and X = L (leucine) or V(valine) and if these interactions may have harmful effects on *in vitro* cultured fibroblasts, as a prototype of mesenchymal cells known to modulate the soft connective tissue environment.

Results obtained by morphological analysis, aggregation kinetics and amyloid-like detection demonstrate that HSs interact with ELPs and through enhanced aggregation kinetics, that HSs differently interact with amyloidogenic ELPs and promote β-transition accelerating amyloid fibril formation and self-association of pre-formed fibrillar structures, as already demonstrated for other proteins^42–44^. These effects were dependent on HSs and ELPs, but independent of the two HS concentrations we have used, as suggested in previous experiments, where the aggregation process reaches a plateau at a certain glycosaminoglycan (GAG)/peptide molar ratio^45,46^.

Generally, the formation of ion pairs between positively charged protein/peptide side chains and negatively charged GAG’s groups is assumed to be the most prominent cause of GAG-protein interactions^24,37,42,44,46,47^ triggering the onset of oligomers and the subsequent formation of mature fibrils. However, this type of interaction may only have a little influence in our experimental conditions, since all peptides are highly hydrophobic and devoid of positive charges except those deriving from the amino groups.

This observation is further supported by ThioflavinT (ThT) experiments performed in Tris buffer solution, where ‘ionic’ interactions exhibit no significant effect on ELP fibrillation indicating that electrostatic interactions do not significantly contribute to the enhancing effect of HS on ELPs fibril formation and lateral aggregation.

It could be therefore suggested that the polymeric nature of HSs exert a template action on ELPs through the combined contribution of van der Waals forces and polar interactions due to the polypeptide backbone, H-bonds involving saccharide hydroxyls and amide groups of the peptides as well as hydrophobic interactions of carbohydrate backbone and N-acetyl groups with aliphatic peptides lateral chains^6,42,47–49^.

These interactions may favour an increase in the local concentration of peptides and/or prefibrillar intermediates, thus promoting rapid fibrillation, and may also explain differences we have observed in the aggregation kinetics and fibrillar morphology induced by natural- and semisynthetic-heparan sulfates (n-HS and ss-HS). Higher molecular weight HS (n-HS) is in fact more able to speed fibrillogenesis than the lower molecular weight HS (ss-HS) possibly through a higher number of interactions between peptide chains and saccharide moieties from n-HS compared to those from ss-HS.

To be noted that interactions with HSs are especially effective on the more hydrophobic (LGGVG)_n_. As already remarked, substitution of the first valine of the sequence with a leucine significantly reduces the amyloidogenic nature of the polymer^31,32^, however, addition of HS significantly increases Congo Red (CR) staining and ThT fluorescence, most likely because of a greater localization of the peptide chains induced by enhanced hydrophobic interactions with leucine side chains.

**Figure 5:**
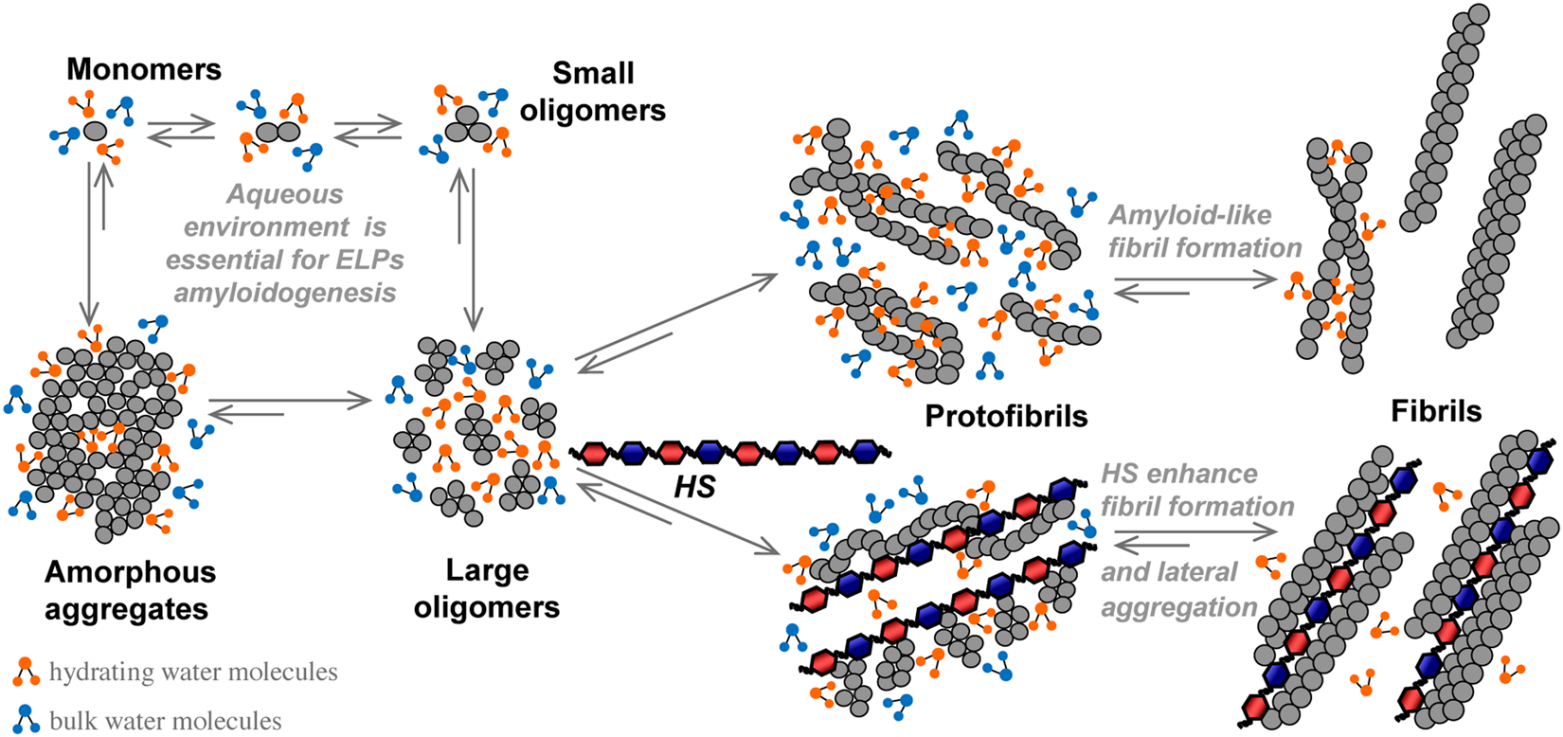
A schematic hypothetical model of HS-mediated enhancement of ELPs self-assembly into amyloid-like fibrils in aqueous environment at 37 °C. Initially, free monomers in solution self-assemble into small and/or large intermediate oligomers and amorphous aggregates acting as nucleation seeds and leading to the formation of protofibrils with different morphology, which merge and intertwine yielding to elongated mature amyloid fibrils. In parallel, higher molecular weight n-HS interact with amyloidogenic intermediates/species more effectively than the lower molecular weight ss-HS. This event can generate a high local concentration of peptides on the HS scaffold that drives the association of amyloidogenic ELPs. Moreover, the hydrophilic nature of HSs may help the β-sheets to associate with one another by sequestering the water molecules through interactions with the disaccharide moieties. The final result of these events combined with the accelerated release of water previously trapped in protofibrils may explain the different acceleration of the overall aggregation pathway leading to amyloid fibril formation, the marked enhancement of their lateral aggregation in more packed fibrillar assemblies as well as the differences in cell adhesion on fibrillar coated surfaces.

Moreover, in these events, it cannot be excluded the critical role of water controlling the self-assembly process of amyloid-like oligomers and protofibrils. Previous studies on (VGGVG)_n_^31,50^ demonstrated that water molecules promote amyloid-like structure through ‘bridging’ hydrogen bonds with the amide groups of antiparallel-β structured peptide chains. The subsequent assembly of the β-sheets requires the water molecules between the sheets to be eliminated concurrently to their reinforcement and extension^32,51^. Therefore, the hydrophilic nature of HSs may help β-sheets to associate with one another by sequestering the water molecules through interactions with the saccharide hydroxyl groups, the final result being the accelerated release of water previously trapped into peptide protofibrils. The process is entropically favoured for peptides rich in hydrophobic residues^51^. Based on previous observations also from other laboratories^51^ it could be proposed a model of interactions between HS and ELP (Fig. 5).

Once it was demonstrated that HSs interact with ELPs, it was mandatory to better understand the biological effects of these interactions. HSs or ELPs, alone or in combination with different HSs, were added to the medium of cultured Balb/c 3T3 fibroblasts. Cell growth and the percentage of cell death did not change compared to optimal standard culture conditions. Moreover, for a preliminary screening of the redox status^52^, we have measured the amount of reactive oxygen species (i.e. hydrogen peroxide and anion superoxide) typically involved in the oxidative damage of biomacromolecules. No changes were observed in all culture conditions.

Results demonstrate that, in solution, this amyloidogenic material, with or without HSs, is not cytotoxic and it does not inhibit cell growth.

However, when these amyloid-like aggregates were used to coat the culture plate surfaces, cell adhesion to these substrates appeared affected. Since serum proteins (i.e. fibronectin and laminin) are known to be absorbed from organic substrates thus favouring cell adhesion and, at the same time, amyloids can mediate cell adhesion by stabilizing and accelerating the deposition of serum proteins^53,54^, in these experiments, a serum-free medium was used. In our experimental conditions, an increase of cellular adhesiveness on amyloid fibrils, in comparison to untreated surfaces, was observed. This finding supports the concept that amyloid directly interacts with cells, independently of serum. Moreover, the presence of HSs, with differences according to the type of HS and to ELPs sequence and length, seems to modify the ability of cells to adhere to amyloid-like fibrillar aggregates. Changes can be explained for instance by the size of morphologically different fibrils and by the presence of more packed fibrillar assemblies of amyloidogenic material forming the surface area available for cell adhesion.

This work provides the first glimpse on the effects of HSs interacting with amyloidogenic ELPs and demonstrates that, at least in the conditions used in the present study: i) HSs, depending on the type of HS (n-HS and ss-HS), modify aggregation kinetics and formation of amyloid-like fibrils as well as their packed fibrillar morphology, ii) these effects depend also on the characteristics of the interacting ELPs, iii) cells proliferate in the presence of fibrillar ELPs and HSs without any evidence of cytotoxic effects, iv) cell adhesion on amyloid-like structures is modulated by the fibrils’ supramolecular organization (Table 3). Results suggest that amyloidogenic ELPs can represent a suitable reductionist approach to better understand how HSs interact with elastin peptides and an innovative model to investigate the role of amyloid fibrils in soft connective tissues. Moreover, since increasing evidence indicates that some amyloid structures are inert or well tolerated by cells, thus proposing their use as scaffolds^55,56^, present data underline the possible role of HS addition in the formulation of matrix-based biomaterials with tunable biological properties.

**Table 3:** Table summarizes the effects of natural (n-HS) and semisynthetic (ss-HS) heparan sulfates added to elastin-like peptides in comparison with elastin-like peptides (ELPs) alone.

		Fibrillization kinetics (ThT)^1^	Amyloid quantification (CR)^1^	Presence of fibrils (TEM)^1^	Cell proliferation (Cell counts)	ROS (H_2_DCF-DA; DH_2_)^1^	Cell adhesion (CV)^1^
**VGGVG**	+n-HS	**—**	**—**	No	**—**	**—**	*
	+ss-HS	**—**	**—**	No	**—**	**—**	*
**(VGGVG)**_**3**_	+n-HS	*	**—**	Yes	**—**	**—**	*
	+ss-HS	***	*	Yes	**—**	**—**	*
**(VGGVG)**_**n**_	+n-HS	****	*	Yes	**—**	**—**	**—**
	+ss-HS	**—**	**	Yes	**—**	**—**	*
**(LGGVG)**_**n**_	+n-HS	*	**	Yes	**—**	**—**	*
	+ss-HS	**—**	****	Yes	**—**	**—**	*

*p < 0,05; **p < 0,01; ***p < 0,001; ****p < 0,0001 of ELP + HS *vs* same ELP alone; “ —” indicates no effect.

^1^ThT (Thioflavin T); CR (Congo Red); TEM (Transmission Electron Microscopy); H_2_DCF-DA (2′,7′-dichlorodihydrofluorescein diacetate); DH_2_ (dihydroethidium); CV (Crystal Violet).

## Methods

### Materials

Chemicals and reagents of analytical grade were purchased from Sigma Aldrich (St. Louis, MA, USA) unless otherwise indicated. Best quality MilliQ water was used for preparation of solutions and buffers, that were filtered through 0.45 μm syringe filter before use.

### Heparan sulfates (HSs)

Two types of HSs were used: natural HS (n-HS) extracted from horse spleen (Mw: 29,5 kDa; SO_3_^−^/COO^−^: 0,8) and semisynthetic HS (ss-HS) obtained from heparin by N-desulfatation followed by N-acetylation (Mw: 10,3 kDa; SO_3_^−^/COO^−^: 1,4) kindly provided by Opocrin S.p.A. Research Laboratories (Corlo, Modena, Italy). These HSs, at concentrations ranging from 5 μg/mL to 100 μg/mL, have been already used for aggregation studies in *in vitro* experiments with elastin derived peptides^6,9^. In the present study, HSs were used at concentrations of 10 μg/mL and 50 μg/mL.

### Peptide synthesis

Experiments were carried out using peptide sequences constituted of the motif XGGVG (X = V or L) as listed in Table S1. Synthesis and characterization of elastin-like peptide (ELP) sequences were performed as previously described^32,33,57,58^.

### Cells

Balb/c 3T3 embryonic mouse fibroblasts were grown in Dulbecco’s Modified Eagle Medium (DMEM) (Gibco, Grand Island, NY, U.S.A.), supplemented with 10% foetal calf serum (FCS) (Gibco)^59^.

### Aggregation kinetics monitored by Thioflavin T (ThT) fluorescence assay

To monitor fibrillation kinetics in real-time, Thioflavin T fluorescence assay has become the primary technique^60,61^.

ELPs (10 μg/mL) and different HSs (10 and 50 μg/mL) solubilized in MilliQ water were incubated alone or in combination at 37 °C without agitation up to 24 h. The fibrillation kinetics were assessed, at intervals of 1, 3, 6, 12, 24 hours by removing 250 μL of each polypeptide from the incubated samples and by adding 250 μl of ThT working solution (50 μM) to a final volume of 500 μL. Fluorescence intensity was monitored at 450 nm excitation and 482 nm emission, after briefly vortexing the mixture in the dark, using an LS-50b spectrofluorimeter (Perkin-Elmer, Waltham, MA, USA) into a 1 cm path length quartz cuvette. Data from three experiments in triplicate were then averaged to provide the final values normalized to the highest fluorescence value set at 100%. The averaged fluorescence values *vs* time were analysed with a procedure of best fit, using a single exponential function^43^ with the exception of the monomer where a linear regression function was applied.

### Supramolecular organization by Transmission Electron Microscopy (TEM)

Natural (n-HS) and semysynthetic (ss-HS) heparan sulfates (10 μg/mL) alone and ELP (10 μg/mL) alone or in the presence of HSs were incubated for 24 h at 37 °C. Negative staining was performed by applying 10 μL of each sample onto formvar- and carbon-coated copper grids. Few drops of 1% uranyl acetate in distilled water were used to increase the contrast and the electron density of the samples. After air drying, grids were observed by transmission electron microscopy (Jeol JEM 1200EX, Jeol, Tokyo, Japan) at 80 keV.

### Amyloid detection and quantification by Congo Red (CR) binding essays

#### Light Microscopy (LM)

For detecting amyloid-like aggregates we used the Congo Red (CR), a dye binding to β-sheet rich structures. Plates were coated overnight at room temperature with the following solutions: i) n-HS or ss-HS (10 μg/cm^2^); ii) elastin-like peptides [(VGGVG) or (VGGVG)_3_ or (VGGVG)_n_ or (LGGVG)_n_] (10 μg/cm^2^); iii) mix of each peptide plus different HSs (10 μg/cm^2^ + 10 μg/cm^2^). Briefly, coated plates were stained for 30 min with a saturating solution of CR (80% EtOH: 20% dH_2_O), dried at room temperature and photographed with a Zeiss Axiophot light microscope. Experiments in triplicate were performed at least three times.

#### Spectroscopic assay

CR dye is also used as a highly sensitive spectrophotometric probe and alternative to ThT for easily detecting amyloid fibril formation^61^. Binding determines a characteristic increase in absorption and red shift in the CR absorption band from 490 to 540 nm. Briefly, all different combinations of aggregated ELPs (10 μg/mL) and HSs (10 μg/mL) were incubated at 37 °C up to 24 h without agitation. CR binding was measured spectrophotometrically at 540 nm. Optical density of samples with CR alone was set at 1 and values from three experiments performed in triplicate were then averaged.

### Cell proliferation and propidium iodide staining

Balb/c 3T3 cells (20 × 10^4^) were plated in 35 mm Petri dishes in a total volume of 2 mL of complete culture medium with all different combinations of aggregated ELPs (10 μg/mL) and HSs (10 μg/mL). Cell proliferation was evaluated after 24, 48, 72 and 96 h by cell count in a haemocytometer (Neubauer). Experiments were performed in duplicate and repeated twice. Moreover, in one separate experiment (in quadruplicate), cell viability was assessed by dye exclusion^62^ using propidium iodide (PI). PI is a fluorescent molecule that is impermeable to cells with an intact plasma membrane, however when cell integrity becomes compromised it gains access to the nucleus where it complexes with DNA rendering the nucleus highly fluorescent. Therefore, after 24, 48, 72 and 96 h of culture, harvested cells were washed with phosphate buffered saline (PBS), centrifuged and pellets resuspended in PI staining solution (10 μg/mL PI in PBS). Samples were kept in this solution at 4 °C protected from light until flow cytometry analyses. 10.000 events for each experimental condition were collected and selective gating based on forward scatter (i.e. cell size) and PI fluorescence parameters made it possible to calculate the percentage of cell death^63^.

### Detection of reactive oxygen species (ROS) by flow cytometry

Balb/c 3T3 cells were cultured in absence or in presence of two HSs or of different ELPs (10 μg/mL) with or without HSs (10 μg/mL). After 24 h, ROS analysis was performed by flow cytometry as previously described^52^. Briefly, intracellular levels of superoxide anion (O_2_^•−^) were estimated by incubating Balb/c 3T3 cells with 1 mM dihydroethidium probe (DH_2_) (Molecular Probes, Eugene, OR, USA), whereas levels of hydrogen peroxide (H_2_O_2_) were evaluated by staining cells with 2′,7′-dichlorodihydrofluorescein diacetate (H_2_DCF-DA) (Molecular Probes). Results were normalized to a negative control (i.e. untreated cells incubated with the probe). Experiments were performed in triplicate and repeated three times.

### Cell adhesion

Bacteriologic plates (plates manufactured from polystyrene, a long carbon chain polymer with benzene rings attached to every other carbon, that represents a very hydrophobic nonwettable polymer to which cells have difficulties to attach) (Falcon, Corning Incorporated Life Sciences, Tewksbury, MA-USA) were coated overnight at room temperature in sterile conditions with the following solutions: i) n-HS (10 μg/cm^2^); ii) ss-HS (10 μg/cm^2^); iii) bovine serum albumin (negative control) (10 μg/cm^2^); iv) aggregates of elastin-like peptides [(VGGVG) or (VGGVG)_3_ or (VGGVG)_n_ or (LGGVG)_n_] (10 μg/cm^2^); v) mix of each peptide with different HSs (10 μg/cm^2^ + 10 μg/cm^2^). Tissue culture plates (plates manufactured from treated polystyrene, i.e. hydrophobic polystyrene surface modified to a more hydrophilic surface using gas-plasma under vacuum favouring a good cell attachment; Falcon) were taken as positive control of cell attachment. An aliquot of 2.5 × 10^5^ cells in 2 mL of serum free DMEM was added to each dish and plates were then incubated at 37 °C for 30, 60, 180 and 360 minutes. Briefly, at each time point, attached cells were stained with crystal violet (0.5% in 20% methanol) and, after washes, the colour retained by cells was eluted by 0.1 M sodium citrate in 50% ethanol (pH 4.2) and read in a spectrophotometer at 540 nm^64^. Optical density (OD) values were related to the highest value obtained by the positive control set at 100%.

Experiments in triplicate were performed at least three times.

### Statistical analysis

Data were analysed with GraphPad Prism software, version 6 for MAC (GraphPad Software, San Diego, CA, USA). For Congo red, one way analysis of variance (ANOVA) for multiple comparison was used. Differences between experimental groups were considered to be significant when the probability value was less than 5%.

Time dependent variations of cellular adhesion were analysed by non linear regression and the function that best fitted with our data was hyperbolic curve. Best fit values, standard errors and degrees of freedom obtained from each condition were compared using ANOVA followed by post hoc Tukey testing.

## Electronic supplementary material

Supplementary Information
