# Supplementary material for: Heparan sulfates facilitate harmless amyloidogenic fibril formation interacting with elastin-like peptides
Source: Sci Rep. 2018 Feb 15;8:3115. doi: 10.1038/s41598-018-21472-0 (PMC5814424; doi:10.1038/s41598-018-21472-0)
Supplement: Supplementary file 1 — Supplementary Information [file 41598_2018_21472_MOESM1_ESM.pdf]

Heparan sulfates facilitate harmless amyloidogenic fibril formation interacting with elastin-like peptides.

Federica Boraldi, Pasquale Moscarelli, Brigida Bochicchio, Antonietta Pepe, Anna M. Salvi, Daniela Quaglino

Supplementary information

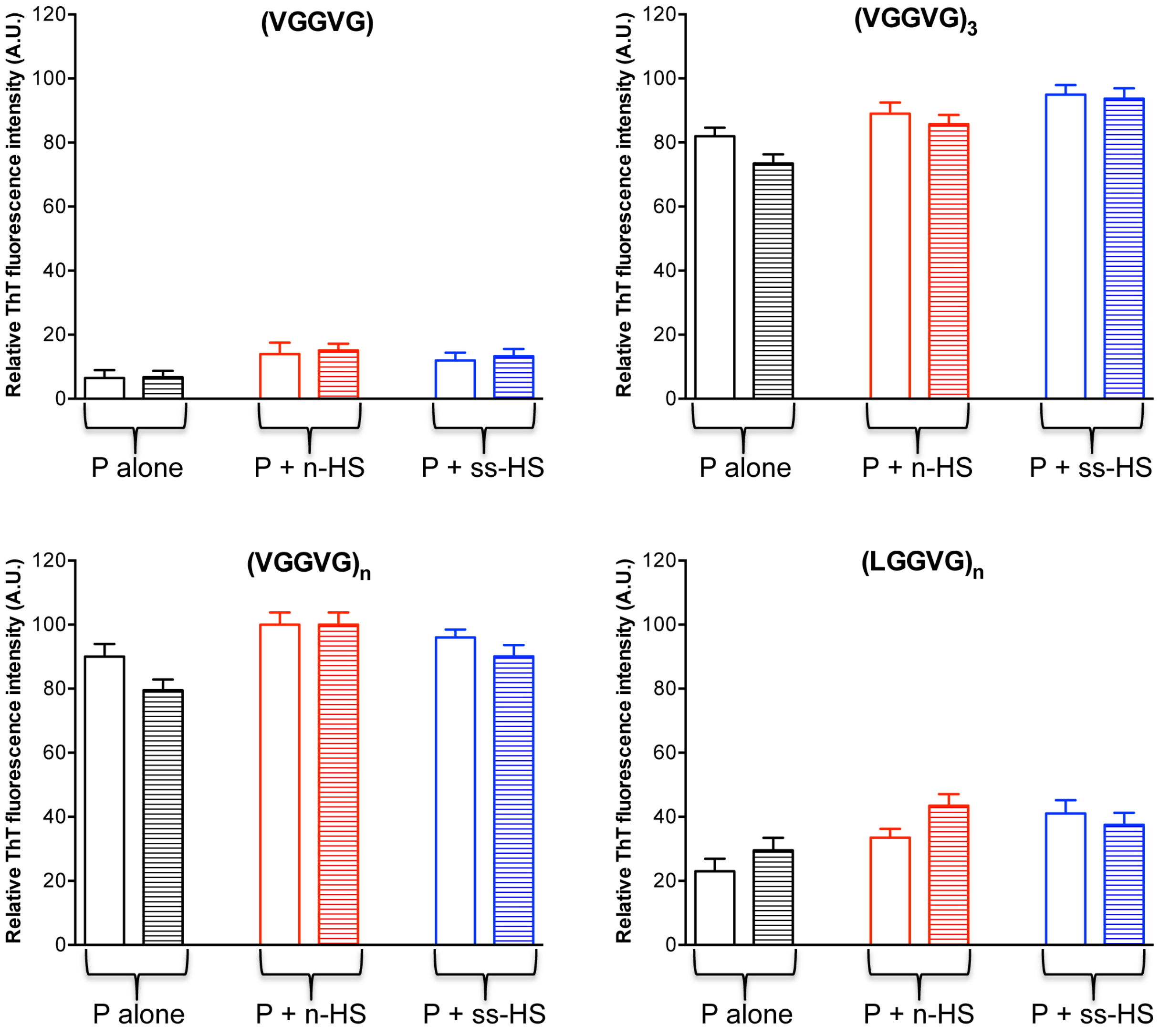

Figure S1. Spectroscopic assay by Congo red (CR). Histograms reveal the amount of amyloid fibrils formed by elastin-like peptide (P) alone or in the presence of natural (n-HS) or semisynthetic (ss-HS) heparan sulfates in aqueous solution (empty bars) or in Tris buffer (solid bars) after an incubation of 24 h at 37°C. Data are normalized to CR background set at 1 and are expressed as the mean ± SD of three experiments.

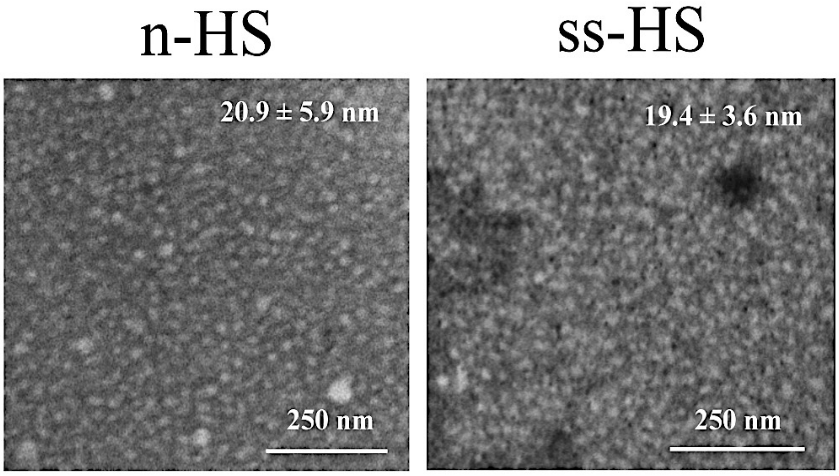

Figure S2. Transmission electron microscopy. Natural (n-HS) and semisynthetic (ss-HS) heparan sulfates in MilliQ water were observed after negative staining. Mean diameter ± SD of HS-forming globular structures is shown.

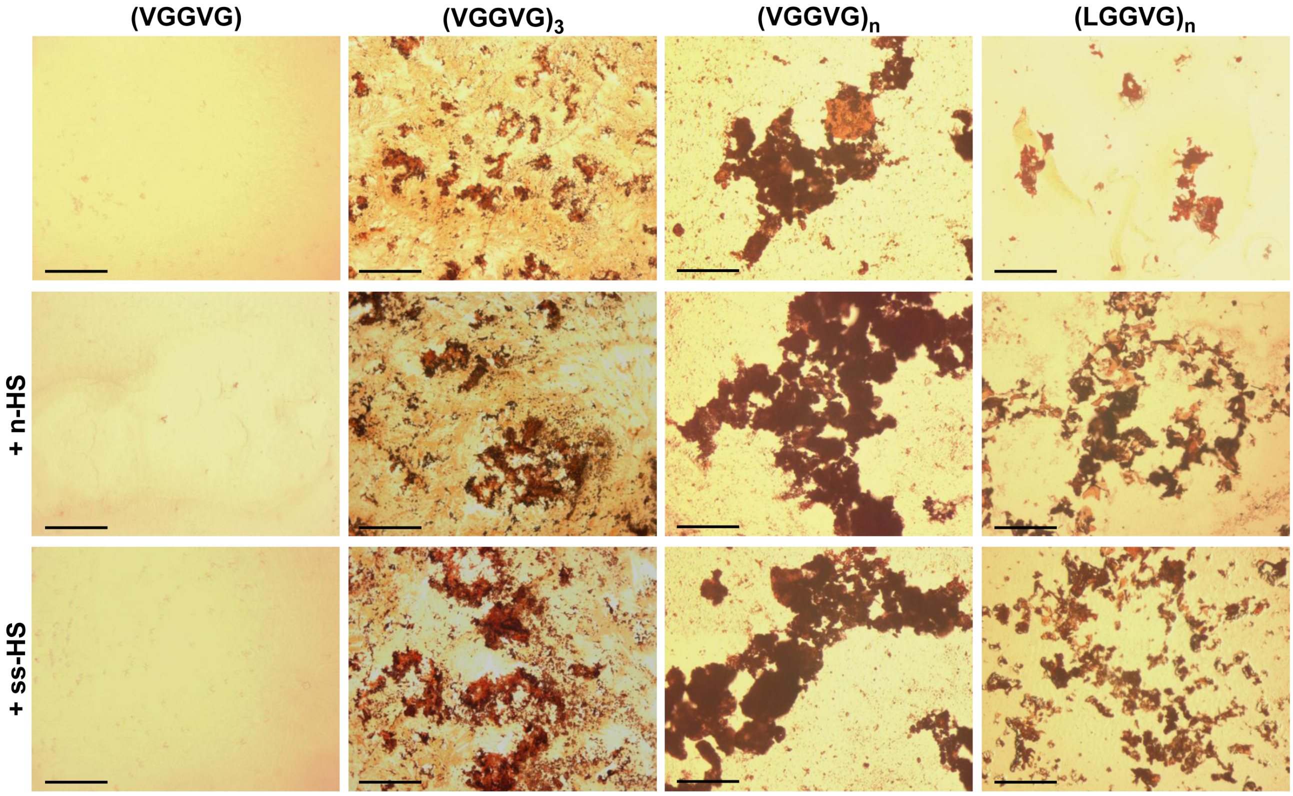

Figure S3. Light microscopy images of elastin-like peptides stained with Congo red dye. Representative images show the stained amyloid-like fibrillar aggregates of different morphologies formed by $(VGGVG)_3$, $(VGGVG)_n$ and $(LGGVG)_n$, in the absence and in the presence of natural (n-HS) or semisynthetic (ss-HS) heparan sulfates in aqueous solution upon incubation for 24 h at 37°C, compared to the (VGGVG) that forms only amorphous aggregates. Bars = 50 μm.

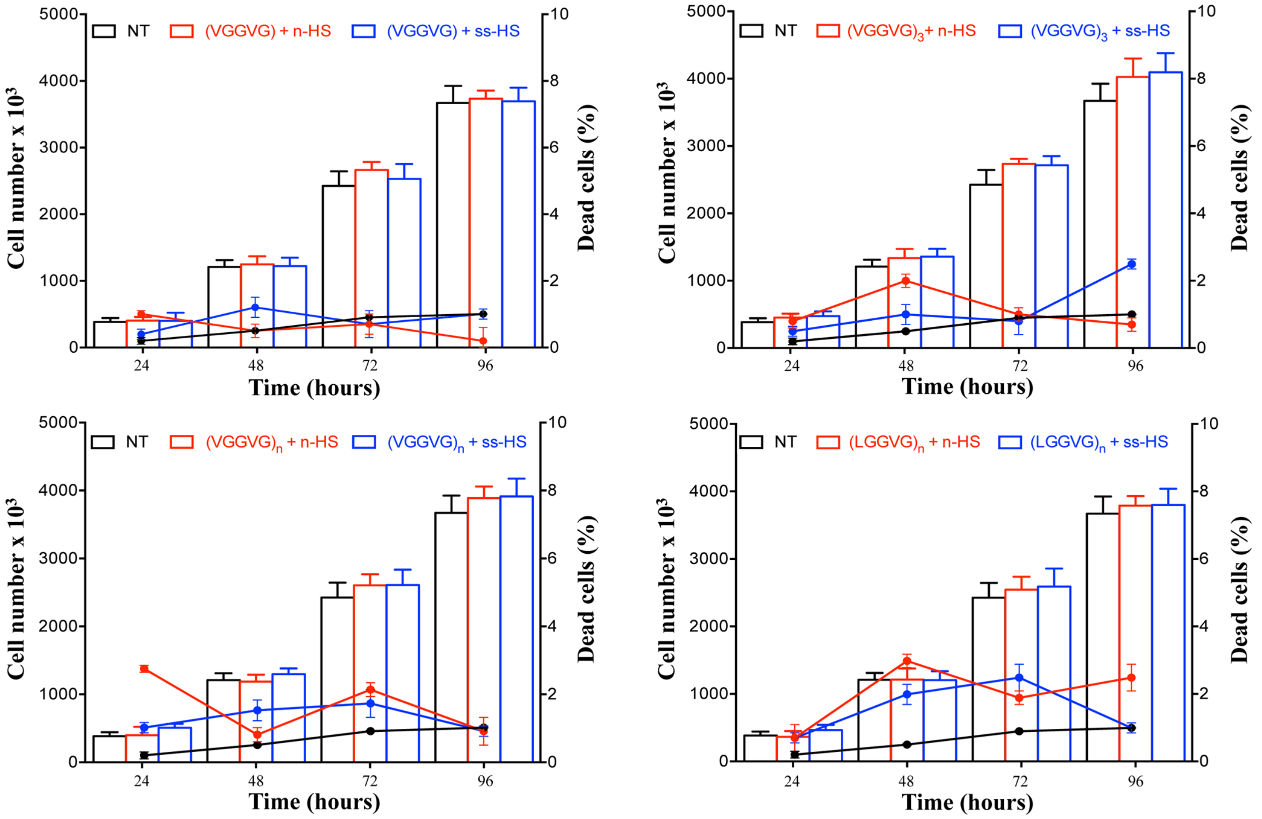

Figure S4. Cell proliferation and cell death evaluations. Comparative observation of cell growth (bars) and dead cells (lines) measured by cell count (left axis) and by the percentage of propidium iodide stained cells (right axis) after 24, 48, 72 and 96 hours of culture in standard medium alone (NT) or supplemented with (VGGVG), $(VGGVG)_3$, $(VGGVG)_n$ and $(LGGVG)_n$ plus natural (n-HS) or semisynthetic (ss-HS) heparan sulfates. Black, red and blue colours indicate cells cultured in NT or in the presence of elastin-like peptide plus n-HS or ss-HS, respectively. Data are expressed as mean values ± SD. Differences were not statistically significant.

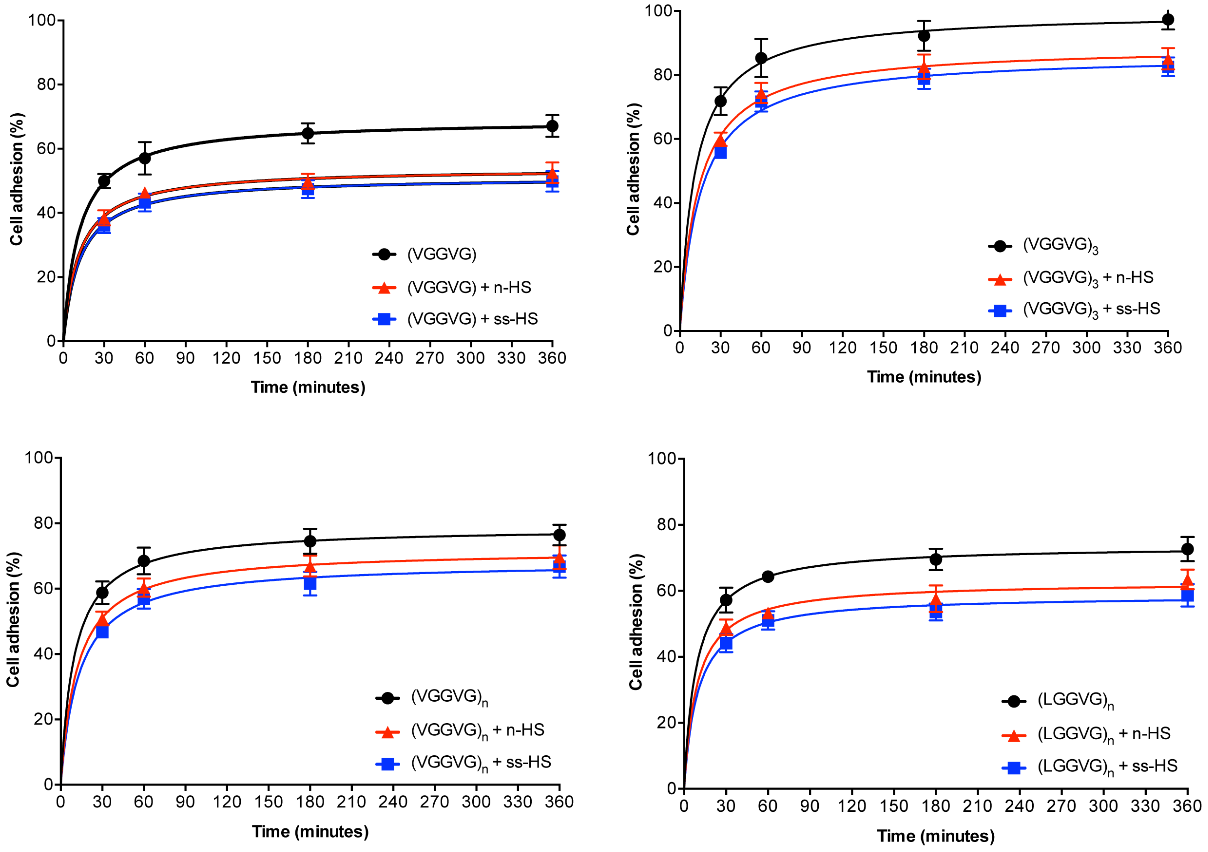

Figure S5. Cell adhesion. At each time point, the percentage of substrate-adherent cells was evaluated by spectrophotometric reading of the crystal violet released by stained cells. Balb/c 3T3 fibroblasts were seeded on surfaces coated with aggregated (VGGVG), $(VGGVG)_3$, $(VGGVG)_n$ and $(LGGVG)_n$ alone or with ELPs plus natural (n-HS) or semisynthetic HS (ss-HS). Values are expressed as percentage of attached cells normalized to the highest attachment value obtained in each experiment by culturing cells on polystyrene tissue culture plates (positive control). Data are represented as mean values ± SD. The hyperbolic curves accurately describe the substrate-adherent cell number – time relationship.

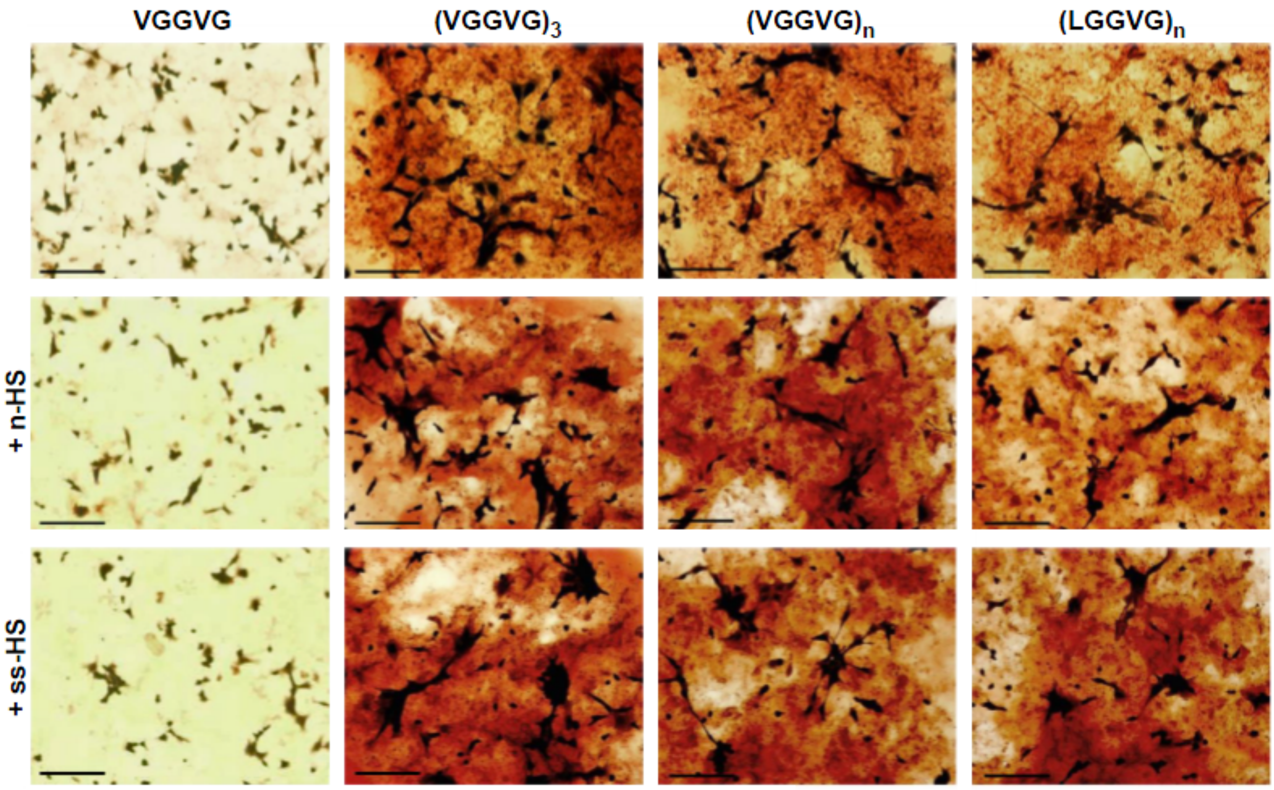

Figure S6. Light microscopy of Balb/c 3T3 fibroblasts grown on Congo red stained substrates. Cells were grown for 6 h in the absence of serum, on coated surfaces with aggregated (VGGVG), $(VGGVG)_3$, $(VGGVG)_n$ and $(LGGVG)_n$ alone or plus natural (n-HS) or semisynthetic (ss-HS) heparan sulfates. Cells (dark brown) and amyloid-like fibrils (dark red) were stained with ferric haematoxylin for 15 min, washed with dH_2O and further stained for 30 min with a saturating solution of Congo red (80% EtOH: 20% dH_2O) and observed by a Zeiss Axiophot optical microscope. Images are representative of at least three experiments done in triplicate. Bars = 50 μm.
